# Neuroprotective effect of liraglutide in an experimental mouse model of multiple sclerosis: role of AMPK/SIRT1 signaling and NLRP3 inflammasome

**DOI:** 10.1007/s10787-022-00956-6

**Published:** 2022-04-01

**Authors:** Reham A. Ammar, Ahmed F. Mohamed, Mohamed M. Kamal, Marwa M. Safar, Noha F. Abdelkader

**Affiliations:** 1 Department of Pharmacology and Biochemistry, Faculty of Pharmacy, The British University in Egypt, Cairo, Egypt; 2 Department of Pharmacology and Toxicology, Faculty of Pharmacy, Cairo University, Kasr El-Aini St., Cairo, 11562 Egypt

**Keywords:** AMPK/SIRT1, Autophagy, Dorsomorphin, Liraglutide, Multiple sclerosis, NLRP3 inflammasome

## Abstract

The heterogeneous nature of multiple sclerosis (MS) and the unavailability of treatments addressing its intricate network and reversing the disease state is yet an area that needs to be elucidated. Liraglutide, a glucagon-like peptide-1 analogue, recently exhibited intriguing potential neuroprotective effects. The currents study investigated its potential effect against mouse model of MS and the possible underlying mechanisms. Demyelination was induced in C57Bl/6 mice by cuprizone (400 mg/kg/day p.o.) for 5 weeks. Animals received either liraglutide (25 nmol/kg/day i.p.) or dorsomorphin, an AMPK inhibitor, (2.5 mg/Kg i.p.) 30 min before the liraglutide dose, for 4 weeks (starting from the second week). Liraglutide improved the behavioral profile in cuprizone-treated mice. Furthermore, it induced the re-myelination process through stimulating oligodendrocyte progenitor cells differentiation via Olig2 transcription activation, reflected by increased myelin basic protein and myelinated nerve fiber percentage. Liraglutide elevated the protein content of p-AMPK and SIRT1, in addition to the autophagy proteins Beclin-1 and LC3B. Liraglutide halted cellular damage as manifested by reduced HMGB1 protein and consequently TLR-4 downregulation, coupled with a decrease in NF-κB. Liraglutide also suppressed NLRP3 transcription. Dorsomorphin pre-administration indicated a possible interplay between AMPK/SIRT1 and NLRP3 inflammasome activation as it partially reversed liraglutide’s effects. Immunohistochemical examination of Iba^+^ microglia emphasized these findings. In conclusion, liraglutide exerts neuroprotection against cuprizone-induced demyelination via anti-inflammatory, autophagic flux activation, NLRP3 inflammasome suppression, and anti-apoptotic mechanisms, possibly mediated, at least in part, via AMPK/SIRT1, autophagy, TLR-4/ NF-κB/NLRP3 signaling.

**Graphical abstract:**

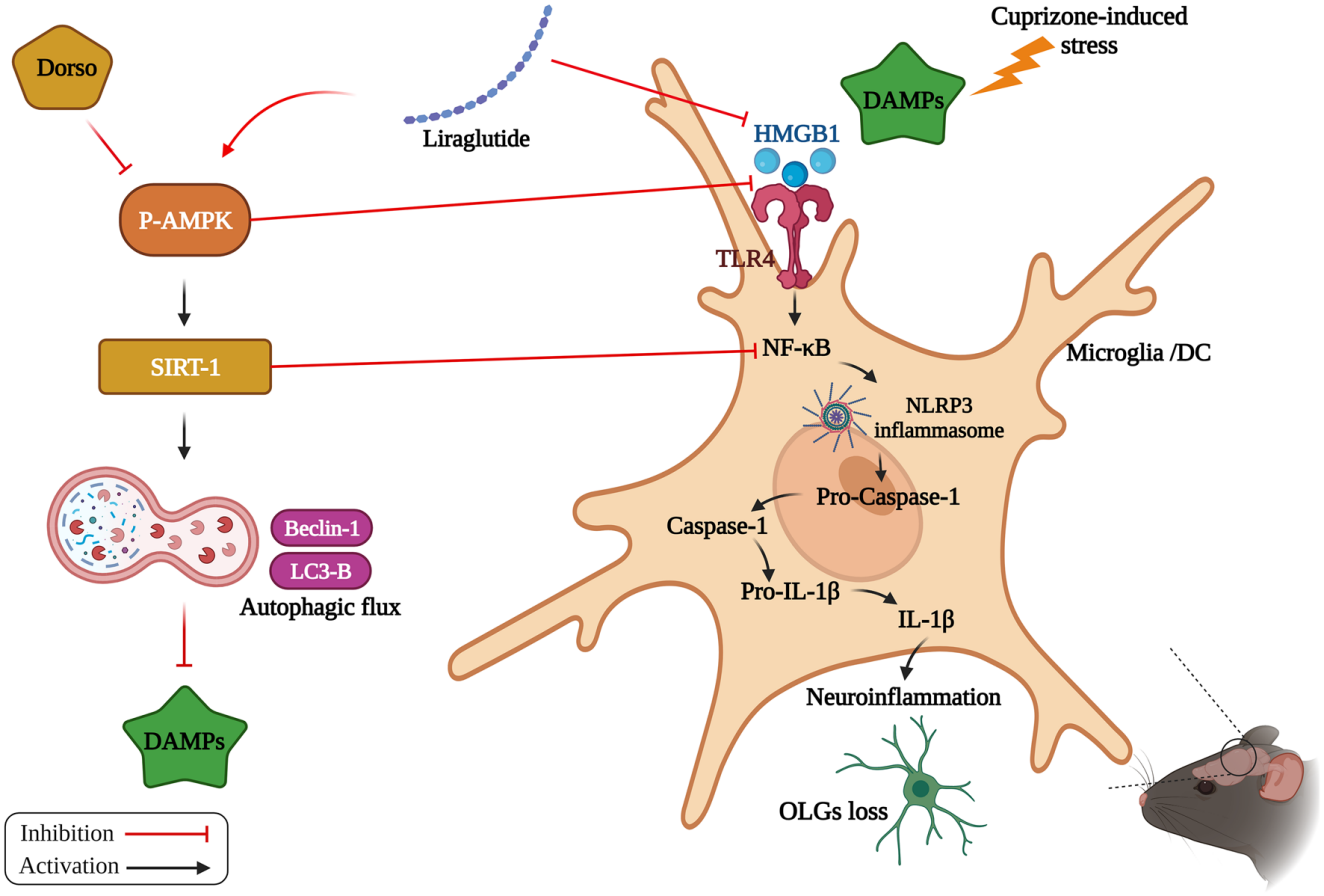

The potential mechanistic insight of Lira in alleviating Cup-induced neurotoxicity via: (1) AMPK/SIRT1 pathways activation resulting in the stimulation of brain autophagy flux (confirmed by lowering Beclin-1 and LC3-B protein expression). (2) Inhibition of NLRP3 inflammasome activation, as evidenced by reduced HMGB1, TLR-4, NF-κB and NLRP3 protein expression, alongside diminishing the activation of its downstream cascade as reflected by reduced levels of caspase-1 and IL-1β protein expression. (3) A possible modulating interplay between the previously mentioned two pathways.

## Introduction

Multiple sclerosis (MS) is a neurodegenerative disease of the central nervous system (CNS) featured by chronic cardinal progressive immune-mediated neuro-inflammatory events that lead to neuronal demyelination, loss of myelin-producing oligodendrocytes (OLGs), and eventually nerve fibers destruction (Khan et al. 2014). Nowadays, MS is manifested as the most prevalent cause of non-traumatic progressive neurological disabilities in youth—about 2.3 million affected individuals globally (Browne et al. 2014)—impairing movement, sensory functions and cognition (Omotoso et al. 2018; Zidan et al. 2018). The currently available treatments mostly target the manipulation of immune response although improving oligodendrocyte progenitor cells (OPCs) differentiation into mature myelin-producing OLGs, which is a crucial step in reversing the degeneration by re-myelination, is an emerging yet elusive therapeutic strategy (Kremer et al. 2016; Plemel et al. 2017).

Sirtuin 1 (SIRT1), a histone deacetylase-Sirtuin family member, is a key modulator of the inflammatory responses. SIRT1 suppresses immunoinflammation via nuclear factor-κB (NF-κB) expression attenuation (Matsushita et al. 2013). The AMP-activated protein kinase (AMPK) is a critical regulator of mitochondrial energy homeostasis (Largani et al. 2019). AMPK exerts anti-inflammatory activity in a SIRT1-dependent manner (Peixoto et al. 2017). Additionally, AMPK and SIRT1 mutually modulate each other showing synergistic activity (Lan et al. 2008). Previous studies elucidated that the AMPK/SIRT1 pathway mediates neuroprotection, partially, through inducing the autophagy machinery hence suppressing cellular damage and apoptotic processes especially via mitochondrial-specific autophagy (mitophagy) (Jin et al. 2014; Yan et al. 2017; Kruppa et al. 2018). In this context, cuprizone (Cup), a copper chelator, has been reported to exert a reproducible neurotoxicity that results from perpetuating mitochondrial energy homeostasis (Zatta et al. 2005) and copper-dependent enzymes inhibition (Messori et al. 2007). Accordingly, it generates a drastic metabolic oxidative stress leading to apoptosis of OLGs, microgliosis and astrogliosis (Hillis et al. 2016).

Nucleotide-binding and oligomerization domain-like receptors-pyrin domain-containing protein 3 (NLRP3) inflammasome is damage motifs sensitive cytosolic protein complex that is expressed by phagocytes including macrophages and dendritic cells (Guo et al. 2015). They are essential part of the protective innate immune response, on the other hand, their over-activation is a key factor in multiple neurodegenerative diseases including MS through copious inflammatory cytokines production including interleukin-1β (IL-1β) and IL-18 (Swanson et al. 2019). IL-1β, a profound mature inflammatory cytokine processed by inflammasomes in microglia, has a pivotal role in cell death and the disturbance of blood–brain barrier integrity (Schrijver et al. 2003; Wang et al. 2014). Noteworthy, targeting NLRP3 inflammasome is an emerging strategy in manipulating MS (Shao et al. 2018).

Data suggest that a cross-talk between AMPK/SIRT1 and NLRP3 inflammasome pathways may occur through NF-κB modulation and/or mitigating mitochondrial damage via mitochondrial reactive oxygen species (mtROS) (Yan et al. 2017; Zou et al. 2018). Furthermore, previous studies revealed that AMPK activation may have a suppressive activity on Toll-like receptor-4 (TLR-4) expression, subsequently attenuating NLRP3 activation (Soraya et al. 2012, 2014). The previous findings cast light on targeting both AMPK/SIRT1 and NLRP3 as an outstanding strategy in the treatment of MS.

Liraglutide (Lira) is a glucagon-like peptide-1 (GLP-1) analogue that is characterized by a high amino acid sequence similarity, 97%, with the human GLP-1. It’s approved for type-2 diabetes mellitus, as a once-daily antidiabetic agent, without hypoglycemic side effects (Jacobsen et al. 2016). Its kinetic properties allow it to pass the blood–brain barrier and subsequently activate the widely distributed GLP-1 receptors (GLP-1R) in the brain (Hunter and Hölscher 2012; McGovern et al. 2012; Cork et al. 2015). Previous studies have reported some neuroprotective effects of Lira in animal models of Alzheimer’s disease (Hansen et al. 2016), Parkinson’s disease (Badawi et al. 2017), cognitive impairment (Liu et al. 2020), cerebral ischemia (Briyal et al. 2014), and diabetic peripheral neuropathy (Moustafa et al. 2018a, b).

The Cup model is a widely used mouse model imitating demyelination–re-myelination events in different brain regions (Klein et al. 2018). Therefore, we hypothesized that Lira has a possible neuroprotective effect against the Cup demyelinating model and a potential to induce re-myelination, suggesting its dual benefit for MS patients with diabetic co-morbidity. Herein, we focused on AMPK/SIRT1 and NLRP3 inflammasome pathways as well as the subsequent defective autophagy, inflammation, and apoptosis.

## Materials and methods

### Animals

This in-vivo study utilized male C57Bl/6 mice (8–10 weeks old, 20–25 g in weight). Mice were kept in the animal facility of the Faculty of Pharmacy, The British University in Egypt (BUE) (Cairo, Egypt) 1 week before the start of the experiment. They were maintained under standard conditions of five mice per cage and 12/12 h light/dark cycle (with lights on at 6 am) at a constant temperature (23 ± 2 °C). Animals were allowed free access to standard chow diet and water ad libitum*.* The experiment followed the guidelines of the animal facility and the experimental protocol was approved by the ethical committees at the British University in Egypt (Approval No. EX-1901, September 2019) and the faculty of pharmacy, Cairo University (Approval No. PT-2421, April 2019).

### Drugs

Cuprizone (Bis (cyclohexanone) Oxaldihydrazone) and dorsomorphin (Dorso) (6-[4-(2-Piperidin-1-ylethoxy) phenyl]-3-pyridin-4-ylpyrazolo [1, 5-a]pyrimidine) were purchased from Sigma–Aldrich Co. USA. Liraglutide was acquired from Novo Nordisk pharmaceutical company, USA. Any other chemical used was of analytical grade with the highest purity.

### Demyelination induction

Cuprizone suspension was prepared by mixing the powder with 0.1% methylcellulose (MC) and vortexed to obtain a homogenous suspension. Animals received Cup (400 mg/kg/day p.o) using oral gavage throughout a period of 5 weeks to induce acute demyelination (Zhen et al. 2017).

### Experimental design and study time scale

Animals were assigned randomly into four groups (*n* = 15); thereafter they were subjected to the following treatments: (i) Normal control group: received 0.1% MC (10 ml/kg p.o.) (Zhen et al. 2017). (ii) Positive control group: received daily oral Cup suspension as described before. (iii) Liraglutide-treated group: received Cup + Lira (25 nmol/kg/day i.p.) for 4 weeks. (iv) Liraglutide and Dorso-treated group: received Cup + Lira + Dorso (2.5 mg/kg i.p.) 30 min right before Lira injection and it was dissolved with normal saline (Lee et al. 2019). A power analysis of power = 0.8 and α = 0.05 was performed to determine the group size with an effect size used previously by Elbaz et al. (2018).

**Fig. 1:**
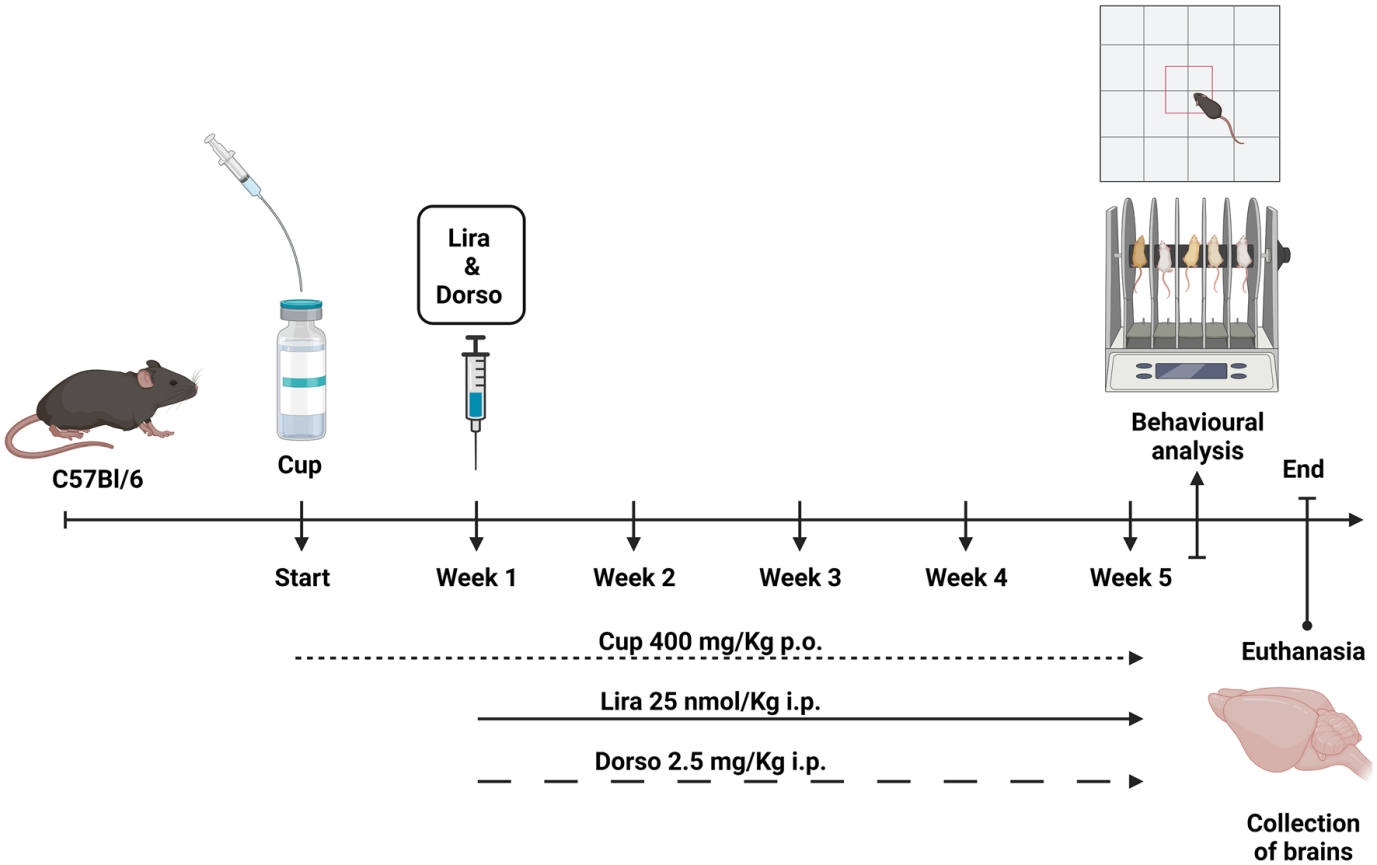
Experimental time scale of Lira and Dorso in Cup-induced demyelination mouse model

Treatment with Lira and Dorso was introduced at the second week of the regimen and continued for 4 weeks. This design is illustrated in Fig. 1. Liraglutide dose has been selected as it was reported to have a neuroprotective effect in previous animal studies (Hunter and Hölscher 2012; McClean and Hölscher 2014).

Behavioral assessments were conducted 24 h after the last dose injection. Eventually, animals were euthanized by decapitation under light anesthesia; afterwards, the whole-brain tissues were rapidly excised and weighed. Within each group, tissues were randomly divided into two subsets: one for histopathological examination and immunohistochemical analysis (*n* = 3) and the other for the investigation of biochemical parameters (*n* = 12).

### Behavioral studies

Animals’ locomotor and motor coordination activities were assessed using open field test (OFT), rotarod and grip strength tests. Worthy of note, behavioral tests were performed with a minimum of 30 min rest interval in between and during animals’ light cycle to avoid any discrepancies due to circadian changes.

#### Open field test

Animals were allocated centrally in a square white wooden box arena, 40 × 40 × 25 cm, illuminated by white dim light, and allowed free uninterrupted movement. The testing apparatus was cleaned using 70% ethanol between subjects to eliminate any bias by left odors. The locomotor activity of each mouse was recorded within a noise-attenuated room for 3 min period, tracked and analyzed via ANY-maze software (version 6.0, Stoelting Co., Wood Dale, USA). The test arena was virtually divided into (4 × 4 cm) 16 equal squares and locomotor parameters including distance traveled, mean speed, mobility time, ambulation frequency (line crossings) and rearing frequency for each animal were assessed (Walsh and Cummins 1976; Keller et al. 2018).

#### Rotarod test

Assessment of motor coordination was performed using a standard rotarod apparatus (rotating at 25 rpm around a 3-cm diameter rod). At first, mice were allowed to acclimatize to maintain their posture on the rotating rod through three 5 min-training sessions on three consecutive days. After assessing the locomotor activity via open field test, animals were placed on the rotating rod and the time each mouse took before falling off the rod was determined as the latency period with a maximum cut-off value of 300 s (Jones and Roberts 2011; Gilli et al. 2016).

#### String test

String test has been used as a method to assess grip strength implicating the neuromuscular function (Brooks and Dunnett 2009; Takeshita et al. 2017). Mice were allowed to hang on a horizontal steel wire (35 cm in length and 2 mm in diameter) by their forelimbs. The latency period through which each mouse was hanging on the wire before losing grip was recorded (Cut-off value 3 min).

### Sample preparation

Brain tissues were collected, rinsed with ice-cold phosphate buffered saline (PBS) to remove any blood residues, and dissected into two hemispheres through the midline. Then, tissues were immersed in liquid nitrogen to snap freeze and stored at − 80 °C till the analysis of the biochemical parameters. Brain hemispheres were homogenized with radioimmunoprecipitation assay (RIPA) buffer (25 mM Tris–HCl pH 7.6, 150 mM NaCl, 1% NP-40, 1% sodium deoxycholate, 0.1% SDS) and protease and phosphatase inhibitor cocktail (Pierce, ThermoFisher Scientific, USA) for protein extraction, and the homogenate was then constantly agitated for 1 h at 4 °C. The mixture was centrifuged for 30 min at 12,000 rpm at 4 °C and the supernatant was aspirated and stored at − 80 °C. Total protein content was quantified using bicinchoninic acid protein quantification kit (ThermoFisher Scientific, USA, Catalogue No. 23225). Protein extracts were used for western blot analysis and enzyme-linked immunosorbent assay (ELISA).

### Biochemical analysis

#### Determination of brain p-AMPK, HMGB1, NF-κB, NLRP3, Caspase-1, and IL-1β via enzyme-linked immunosorbent assay

Mouse p-AMPK (Thr 172) (Catalogue No. MBS7230575, My BioSource, USA), NF-κB (P65) (Catalogue No. MBS775083, My BioSource, USA), high mobility group box protein-1 (HMGB1, catalogue No. CSB-E08224r, CUSABIO Technology LLC, China,), NLRP3 (Catalogue No. LS-F39627, LSBio, USA), caspase-1 (Catalogue No. NBP2-75014, Novus Biologicals, USA) and IL-1β (Catalogue No. MLB00C, R&D systems, USA) protein expression were determined and quantified using ELISA kits according to the manufacture’s protocol.

#### Western blot analysis of SIRT1, TLR-4, MBP, Olig2, and autophagy biomarkers (Beclin-1 and LC3B) protein expression

Equal amounts of protein samples (30 μg) were allowed to separate on 4–20% Precast polyacrylamide (SDS–PAGE) gels (ThermoFisher Scientific, USA). Thereafter, into a nitrocellulose membranes (Optitran^®^, Whatman), the separated proteins were transferred. Following the transfer, the nitrocellulose membranes were washed with 1X Tris Buffered Saline (1X TBS) and then blocked with 3% milk in 1X TBS for 1 h at room temperature. Subsequently, membranes were incubated overnight at 4 °C with the recommended dilutions of primary antibodies against SIRT1 (Catalogue No. MA5-32912), myelin basic protein (MBP, Catalogue No. MA5-27818), Olig2 (Catalogue No. P21954), Beclin-1 (Catalogue No. PA5-20171), and LC-3B (Catalogue No. PA5-85081) from ThermoFisher Scientific (USA) as well as TLR-4 (Catalogue No. 14358S) and GAPDH (Catalogue No. 2118) from Cell Signaling (USA). After washing with 0.1% tween in TBS, membranes were incubated with the corresponding peroxidase-labeled secondary antibodies at room temperature for 1 h. Finally, the protein-antibody complexes were visualized using enhanced chemiluminescent horseradish peroxidase (HRP) substrate (SuperSignal West Pico plus Chemiluminescent, ThermoFisher Scientific, USA) by scanning the membrane for chemiluminescent bands at the expected molecular weight of the protein via C-DIGIT^®^ blot Scanner (Li-Cor Biosciences, USA). The protein bands density was finally analyzed using Image studio digits software version 5.0 (Li-Cor Biosciences, USA) and the results were then normalized with GAPDH protein expression.

### Histopathological examination

Using 10% neutral buffered formalin, whole-brain tissues were fixed for 72 h. Afterwards, brain tissue samples were processed in serial dilutions of ethanol (100–95%), cleared in xylene and embedded into Paraplast tissue embedding media. Five-micron thick sagittal brain sections were cut from the paraffin tissue blocks by rotatory microtome. Then, the processed tissue sections were deparaffinized and stained with Harris Hematoxylin and Eosin (H&E) stain for general tissue examination, in addition to Luxol fast blue (LFB) for evaluation of myelinated nerve fibers percentage in corpus callosum regions from different samples of all groups. Samples fixation and processing were applied according to (Culling 2013). The histopathological examination was performed by an experienced investigator who was blinded to samples’ identity to eliminate any bias in the results.

### Immunohistochemical assessment of Iba^+^ microglial cells

Unstained deparaffinized 5 μm-thick brain tissue sections were cut and processed with 3% hydrogen peroxide for 20 min, washed by PBS, and incubated with ionized calcium-binding adaptor molecule 1 (Iba-1) primary antibody (Abcam, USA, Catalogue No. ab108539) overnight at 4 °C after 1:100 dilution. HRP performed complex and 3,3̀-diaminobenzidine (DAB) were used for detection of Iba^+^ microglia according to manufacturer’s instructions (Dako, Denmark). After washing by PBS, tissue slides were counter-stained with hematoxylin for microscopic analysis. Micrographs were captured by Full HD microscopic camera processed by Leica application module (Leica Microsystems GmbH, Wetzlar, Germany). The immunohistochemical examination was performed by an experienced investigator who was blinded to samples’ identity to eliminate any bias in the results.

### Statistical analysis

A Shapiro–Wilk test was performed to check for the normality of distribution of all parametric data sets. Data that passed the requirements for parametric criteria were then analyzed by one-way analysis of variance (one-way ANOVA) followed by Tukey–Kramer posts hoc statistical tests and presented as mean ± standard deviation (S.D.). However, data sets that did not meet the parametric requirements were analyzed using non-parametric Kruskal–Wallis ANOVA followed by Dunn’s post hoc statistical tests and presented as median and range. Data were analyzed and visualized using GraphPad Prism software (Version 9, San Diego, CA, USA). A cut-off value of less than 0.05 was accepted as a statistically significant *P* value level. Additionally, to confirm that, sample sizes had the sufficient power to detect a statistically significant effect, Mead’s “Resource Equation” was applied (Mead et al. 2012).

## Results

### Lira ameliorated cuprizone-induced behavioral abnormalities

**Fig. 2:**
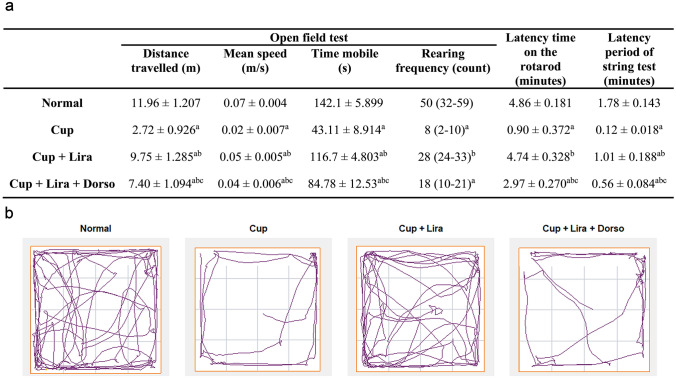
Effect of Lira on Cup-induced alterations in locomotion & motor ability in C57Bl/6 mice through OFT, rotarod and string test. **a** Table of the behavioural analysis results. Each value represents the mean ± S.D. except for the rearing frequency, values represent the median and range of 12 mice in the open field arena. **b** Represents track plots showing the position of the centre of the mice in OFT test. Statistical significance between groups was detected using one-way ANOVA followed by Tukey–Kramer post hoc tests except for rearing frequency, which was analyzed using Kruskal–Wallis ANOVA followed by Dunn’s post hoc statistical tests. ^a^Significantly different from Normal group, ^b^Significantly different from Cup group, ^c^Significantly different from Cup + Lira group at *P* < 0.05

Behavioral tests demonstrated that Cup administration resulted in a significant deterioration in the locomotor activity and motor coordination parameters compared to the normal control group as displayed in Fig. 2a & b. Treatment with Lira significantly reversed the behavioral changes as it depicted an increase in the total distance traveled by mice, mean speed, time being mobile, and rearing frequency (OFT test) reaching levels approximately 4-, 3-, 3- and 4-fold, respectively, greater than those in the cuprizone-treated group. Interestingly, Lira increased the retention time of animals on the rotating rod before falling (rotarod test) and the latency period the mice took hanging on the steel wire (string test) reaching approximately 5- and 8-fold the cuprizone-treated group values, respectively, compared to the Cup-treated group. Meanwhile, combining Lira with Dorso caused worsening in the behavioral parameters compared to the Lira-treated group.

### Lira reversed cuprizone-induced brain weight alterations

**Fig. 3:**
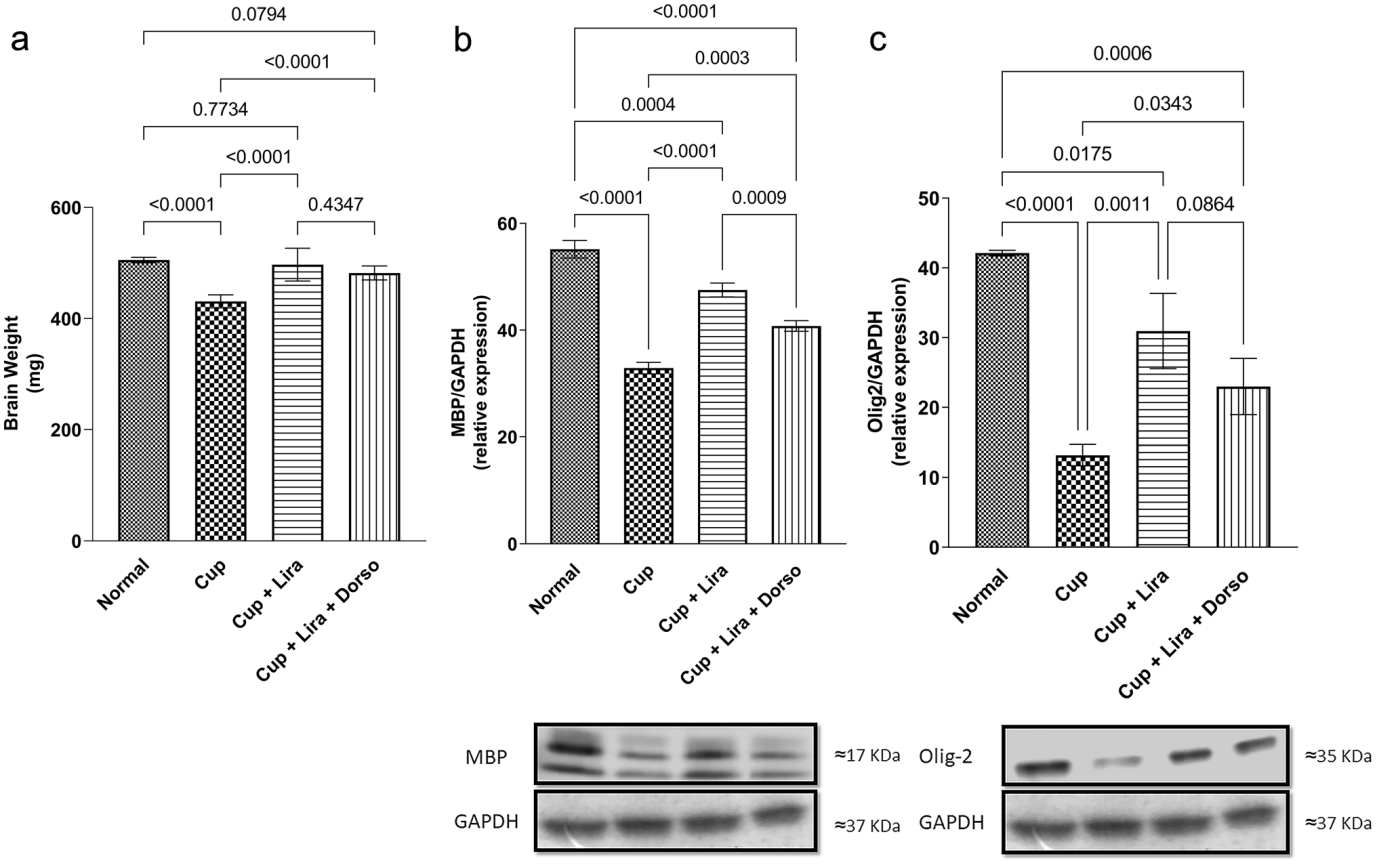
Effect of Lira on Cup-induced alterations in brain weight and brain myelination biomarkers of C57Bl/6 mice. Vertical bars represent the mean ± S.D. of 12 mice for **a** brain weight, as well as six mice for **b** MBP and **c** Olig2 relative protein expression. Values are statistically significant at *P* < 0.05 using one-way ANOVA followed by Tukey–Kramer post hoc tests

As shown in Fig. 3a, a significant reduction of 14.7% in brain weight was induced by Cup administration in comparison to normal control group. Whereas, Lira significantly increased the brain weight by 15% compared to the Cup group and almost maintained the brain weight compared to normal group. Combining Lira with Dorso decreased brain weight by 3% compared to Lira-treated group.

### Lira improved cuprizone-induced alteration in the brain myelination status.

The percent of myelinated nerve fibers in different corpus callosum fields as well as MBP and Olig2 relative protein expression were measured to assess the effect of Lira administration on C57Bl/6 mice brain myelination status.

**Fig. 4:**
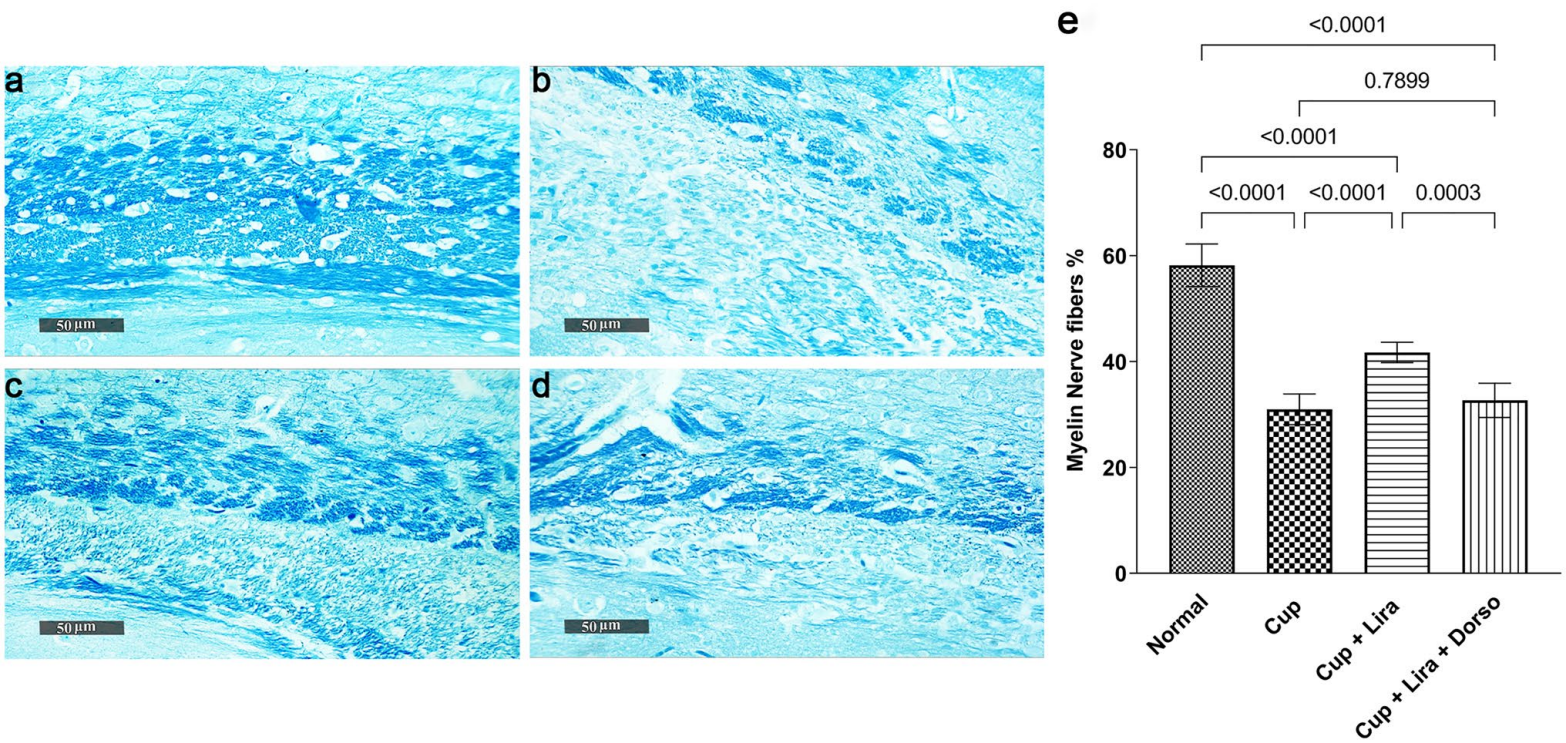
Representative images showing the effect of Lira on Cup-induced deterioration in myelinated nerve fibers percentage. LFB-stained brain corpus callosum coronal sections (magnification, × 50) of: **a** Normal control group, **b** Cup-, **c** Lira-, **d** Lira + Dorso-treated groups LFB-staining intensities and **e** Bar chart for the percentage of myelinated nerve fibres in six random corpus callosum fields/tissue section. Vertical bars represent the mean ± S.D. of each group. Values are statistically significant at *P* < 0.05 using one-way ANOVA followed by Tukey–Kramer post hoc tests

As shown in Fig. 4, Cup administration significantly evoked a defect in the myelination status of mice brain tissues, which is evidenced by the significant decrease in the percentage of myelinated nerve fibers in different corpus callosum brain sections by 46.7%, as depicted by LFB-staining. In contrast, Lira treatment significantly increased the intensity of LFB-staining by 35% and consequently increased the percentage of myelinated nerve fibers in comparison to the Cup group. Worthy of note, Dorso co-administration reduced the percentage of myelinated nerve fibers in comparison to Lira-treated group.

Fig. 3a and b also shows that Lira administration significantly improved the myelination markers in C57Bl/6 mice brains as manifested by the increased levels of MBP and Olig2 relative protein expression reaching approximately 1.5- and 2-fold, respectively, compared to the Cup group. Interestingly, by combining Lira + Dorso, MBP protein expression significantly decreased, while it showed almost the same level of Olig2 in comparison to the Lira-treated group.

### Lira activated AMPK/SIRT1 pathway in cuprizone-induced neurotoxicity

**Fig. 5:**
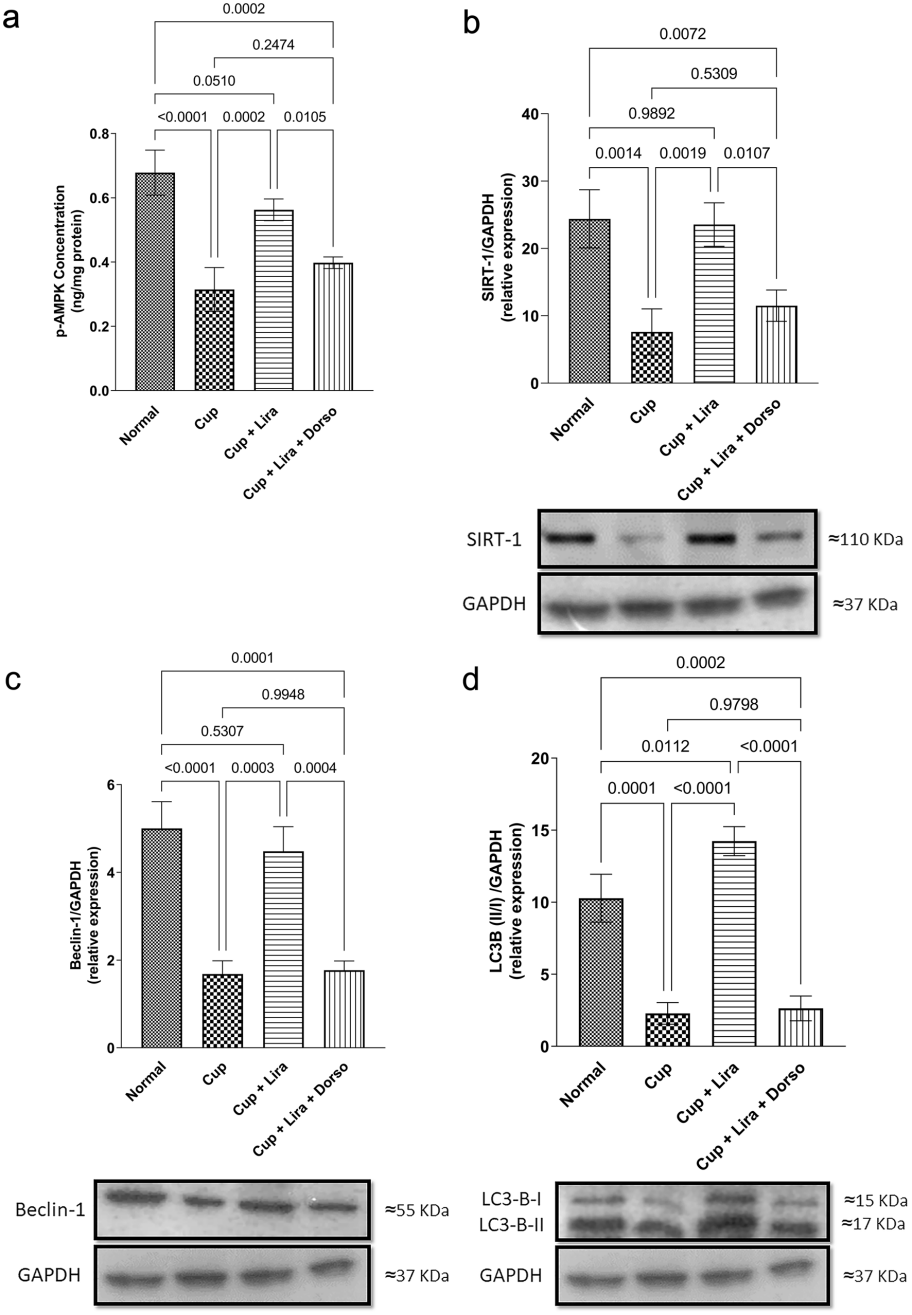
Effect of Lira on brain p-AMPK/SIRT1 and autophagy biomarkers in Cup-induced demyelination in C57Bl/6 mice. Vertical bars represent the mean ± S.D. of six mice for **a** p-AMPK level, **b** SIRT1, **c** Beclin-1 and **d** LC3B (II/I) relative protein expression. Values are statistically significant at *P* < 0.05 using one-way ANOVA followed by Tukey–Kramer post hoc tests

Fig. 5a and b presents the experimental data where Cup administration showed a marked reduction in p-AMPK and SIRT1 levels by 53.6 and 68.8%, respectively, compared to normal control group. Interestingly, Lira almost normalized p-AMPK and SIRT1, when compared with normal control group. In addition, it caused a significant augmentation in both proteins expression reaching approximately 2- and 3-fold, respectively, when compared to the Cup-treated group. On the other hand, combining Lira + Dorso was able to partially block Lira-induced stimulation of p-AMPK/SIRT1 proteins as confirmed by the significant reduction of p-AMPK and SIRT1 by 28.9 and 51.1%, respectively, when compared to Lira-treated rats.

### Lira augmented autophagic flux in cuprizone-induced demyelination

To investigate the effect of Lira administration on C57Bl/6 mice brain autophagy, Beclin-1 and LC3B proteins expression was measured. As presented in Fig. 5c & d, Cup administration inhibited the autophagy pathway as manifested by the reduction of Beclin-1 and LC3B-B (II/I ratio) proteins expression by nearly 2- and 3.3-fold, respectively, compared to normal control group. Lira treatment augmented the brain autophagy process as evidenced by the significant increase in the protein expression of both Beclin-1 and LC3B (II/I ratio) reaching levels approximately 3- and 6-fold, respectively, when compared to the Cup group. Meanwhile, co-administration of Dorso with Lira gave rise to a defect in brain autophagy, a finding that was confirmed by a concomitant decrease in both Beclin-1 and LC3B-B (II/I ratio) protein expression by 1.5- and 4.4-fold compared to Lira-treated group.

### Lira inhibited NLRP3 inflammasome activation and OLGs apoptotic biomarkers in cuprizone-induced neurotoxicity

**Fig. 6:**
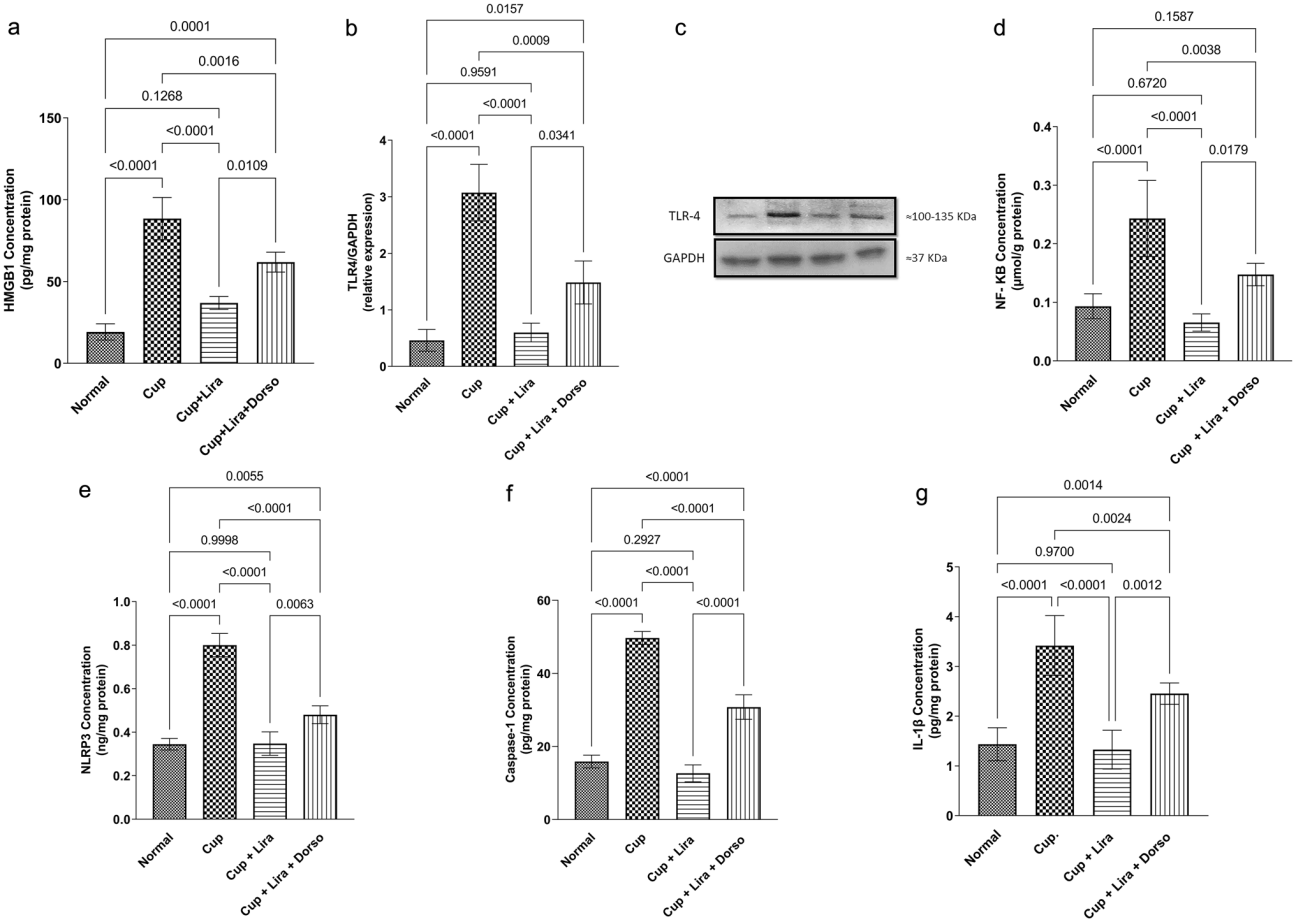
Effect of Lira on Cup-induced inflammation and apoptosis via NLRP3 inflammasome activation in C57Bl/6 mice. Vertical bars represent the mean ± S.D. of six mice for **a** HMGB1, **b** & **c** TLR-4, **d** NF-κB, **e** NLRP3, **f** caspase-1 and **g** IL-1β protein expression. Values are statistically significant at *P* < 0.05 using one-way ANOVA followed by Tukey–Kramer post hoc tests

Cuprizone intoxication significantly evoked the protein expression of HMBG1 and upregulation of TLR-4, hence increased the expression of NF-κB (Fig. 6). Consequently, the NLRP3 downstream cascade proteins were highly expressed. Also, Cup augmented the apoptotic flux as evidenced by increasing the expression of caspase-1 and IL-1β by 2- and 1.5-fold, respectively, compared to normal mice. On the other hand, Lira was capable of curtaining the inflammation induced by Cup ingestion via NLRP3 inflammasome. In the Lira-treated group, HMGB1, TLR-4, NF-κB and NLRP3 proteins expression were decreased by 1.4-, 4.1-, 3- and 1.3-fold, respectively, compared to the Cup-treated group. Furthermore, Lira exhibited a suppression of the apoptotic process as depicted by the significant decrease in apoptotic biomarkers caspase-1 and IL-1β by 3- and 1.6-fold, respectively, less than that observed in Cup-treated group.

### Histopathologic evaluation of different brain coronal sections

**Fig. 7:**
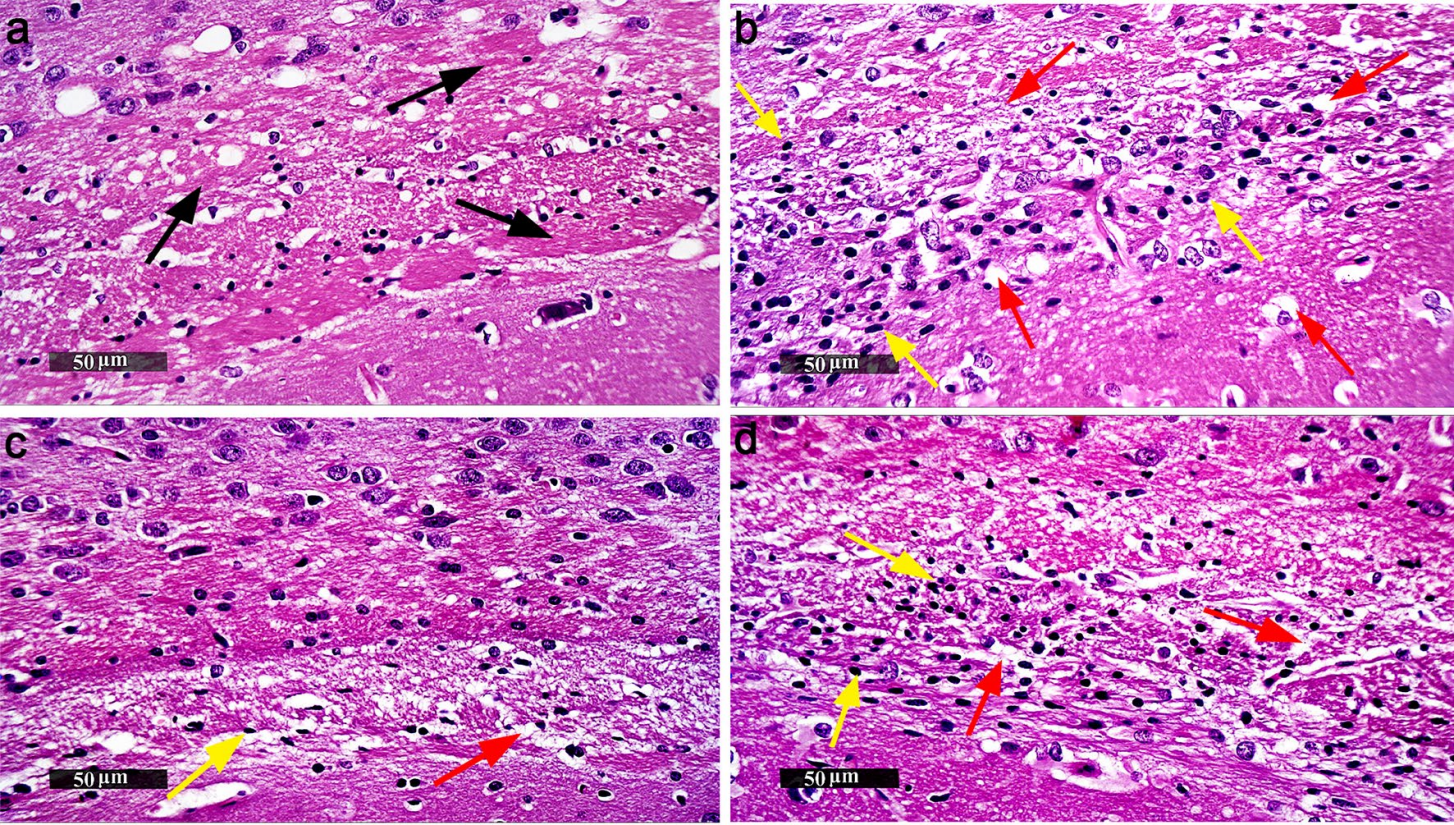
Effect of Lira on histopathological alterations in Cup-induced neurotoxicity. Representative images of H&E stained brain corpus callosum sections of C57Bl/6 mice, (magnification, × 50). **a** Normal control group, **b** Cup-, **c** Lira- and **d** Lira + Dorso-treated groups. Black arrow indicates abnormal glial cells infiltrates, yellow arrow indicates astroglial and microglial cells infiltrates and red arrow indicates demyelination, vacuolization and axonal damage

According to Fig. 7, corpus callosum sections of normal control mice showed normal histological structures with normal myelinated nerve fibers and minimal abnormal glial cells infiltrates. The administration of Cup elicited focal regions of reactive astroglial and microglial cells infiltrates. Moreover, it showed moderate demyelination, vacuolization and axonal damage showing higher inter-axonal spaces all-over corpus callosum. Lira-treated corpus callosum sections presented a significant reduction in glial cells infiltrates with mild focal axonal demyelination. However, most of the fields showed intact and more organized morphological features. On the other hand, sections from Lira + Dorso-treated mice demonstrated almost the same histological records as Cup-treated sections.

### Lira ameliorated cuprizone-induced microgliosis

**Fig. 8:**
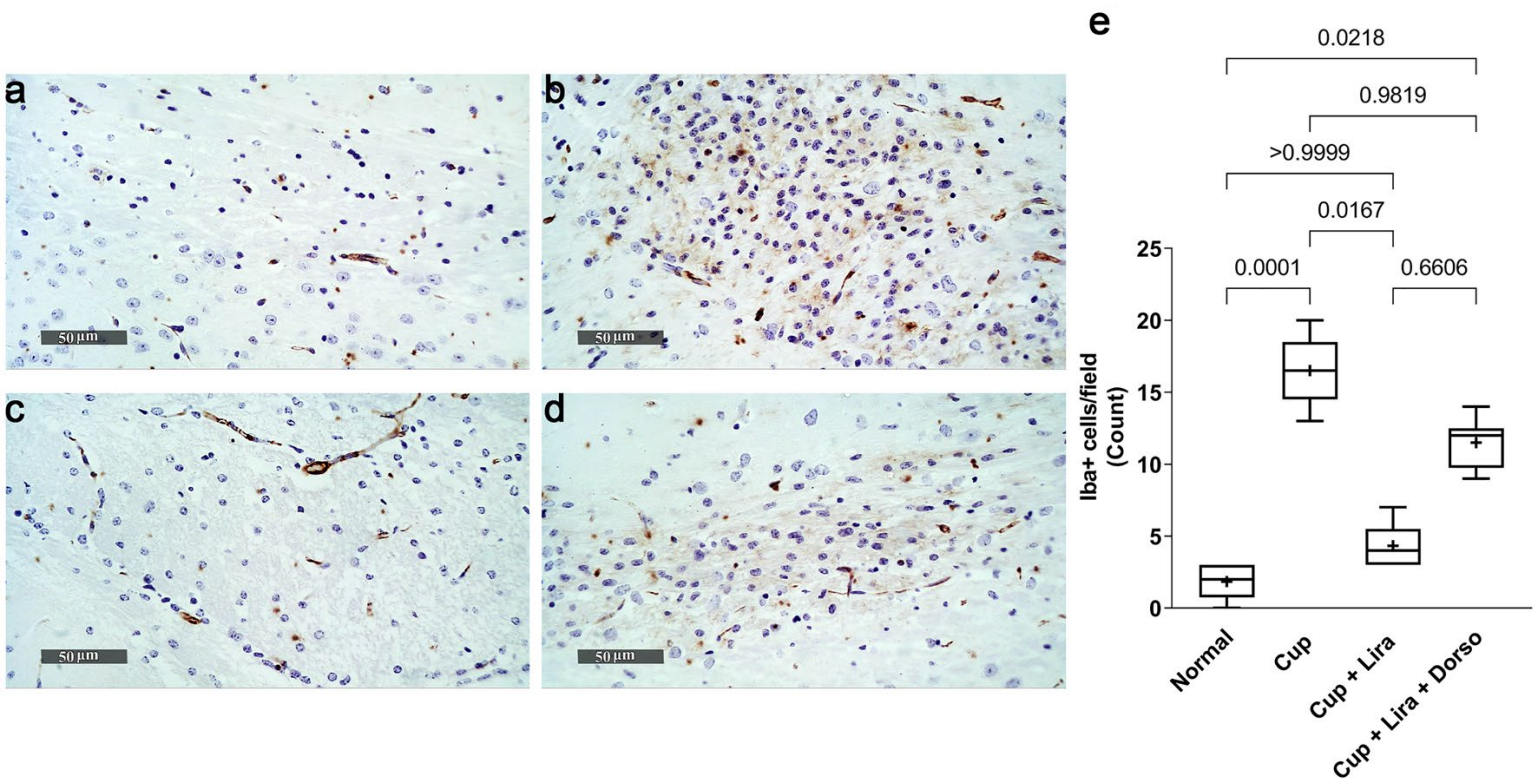
Effect of Lira on Cup-induced microgliosis. Representative images of anti-Iba-1 Immunohistochemical staining of brain corpus callosum sections of C57Bl/6 mice (magnification, × 50). **a** Normal control group, **b** Cup-, **c** Lira-, **d** Lira + Dorso-treated groups, and **e** Box plot chart for the count of active microglial cells that positively express Iba-1 in six random corpus callosum fields per tissue section. Vertical bars represent the median and range of each group. ( +) represents the mean. Values are statistically significant at *P* < 0.05 using Kruskal–Wallis ANOVA followed by Dunn’s post hoc statistical tests

To investigate the relative activation of microglial cells in brain corpus callosum sections, Anti-Iba-1 immunohistochemical staining was performed. According to Fig. 8, Cup-treated sections showed an elevated Iba-1 positive staining reaching approximately 8-fold compared to normal control. Interestingly, treatment with Lira was able to reduce the Iba^+^ stained microglial cells by 73.7% compared to the Cup-treated group. However, combing Lira + Dorso caused an increase in the number of Iba-1 positively stained microglia reaching 3-fold compared to the Lira-only treated group.

## Discussion

The current study divulged, for the first time, new mechanistic insights of the ameliorative effects of Lira on Cup-induced demyelination through the modulation of AMPK/SIRT1, autophagy flux, and NLRP3 inflammasome neuro-inflammatory pathways, and its potential effect to trigger neuronal re-myelination via OPCs differentiation induction (Greenberg and Jin 2006). Dorsomorphin, a pyrazolopyrimidine derivative immensely used as an AMPK inhibitor (Zhou et al. 2001), was used in the current work to investigate the involvement of P-AMPK/SIRT1 signaling in Lira-induced autophagy. Herein, Dorso was not able to abolish the neuroprotective effects of Lira completely, which implies that the neuroprotective effects of Lira involve not only AMPK-dependent but also AMPK-independent mechanisms.

There are several lines of evidence supporting the crucial role of GLP-1R in the growth and vitality of neuronal cells (Duarte et al. 2013; Hölscher 2014; Yildirim Simsir et al. 2018; Elbassuoni and Ahmed 2019). Moreover, GLP-1 knock-out mice showed impaired neuronal functions such as cognition, learning, and memory (Perry et al. 2003; Abbas et al. 2009). It was reported that GLP-1 has a pivotal effect in the stimulation of neuronal progenitor cells proliferation (During et al. 2003), an effect that is beneficial for the restoration of neuronal networks in different CNS diseases such as Alzheimer’s (Greenberg and Jin 2006; Hansen et al. 2016) and Parkinson’s diseases (Badawi et al. 2017; Safar et al. 2021). Indeed, the aforementioned data cast light on GLP-1R as an emerging potential target of new drug discovery in the treatment of MS.

Our findings depicted that, Cup was able to induce multiple behavioral and motor dysfunctions as observed in OFT, rotarod and string testing. These results are explained by the observed abnormally affected corpus callosum regions, in parallel to previous findings (Zhen et al. 2017; Zhang et al. 2020). Whereas, treatment with Lira reversed the impairments in animals’ behavioral profile in addition to histopathological abnormalities. Moreover, the findings were accompanied by a significant Cup-induced decrease in brain weight, which might be attributed to the reported ability of Cup to induce neuronal demyelination through the perturbation of brain mitochondrial metabolic state in mice (Matsushima and Morell 2001; Liu et al. 2010). Whereas, treatment with Lira amended Cup-induced reduction in brain weight, suggesting its potential repurposing in the treatment of MS. Moreover, Dorso has partially blocked the effect of Lira on motor and behavioral dysfunctions, as mice received Dorso showed better behavioral profiles but to a lesser extent than Lira-treated mice. On the other hand, Dorso failed to reverse Lira-induced normalized brain weight.

Myelin basic protein, is a main intrinsic component of the mature compacted myelin sheath covering the neuronal axons (Stinissen et al. 1998). It is well established as an indicative biomarker for the assessment of myelination status and re-myelination tendency (García-León et al. 2020). Herein, Cup administration caused demyelination as manifested by the profound reduction in MBP expression and hence myelin sheath loss, the major hallmark of MS, which lends support to previous reports (Clarner et al. 2012; Abd El Aziz et al. 2021). This finding is affirmed by LFB-staining of brain corpus callosum showing a reduced percentage of myelinated nerve fibers. Promisingly, the present study reveals the potential of Lira to hinder neuronal demyelination and its ability to stimulate re-myelination as Lira-treated group significantly restored Cup-deranged level of MBP sections (Carriel et al. 2017). Additionally, Lira treatment elevated the percentage of myelinated nerve fibers in brain corpus callosum sections. Furthermore, Dorso abated the effect of Lira treatment on myelination status as it showed a lower MBP level than the Lira-treated group, and almost blocked the protective effect of Lira on myelinated nerve fibers percent.

In parallel with re-myelination, Olig2, a master transcription factor, is believed to have a pivotal role in OPCs differentiation into mature myelin-producing OLGs (Szu et al. 2021). According to our results, Cup ingestion reduced OPCs re-myelination capacity through lowering Olig2 protein level, which emphasize previous findings (Zhan et al. 2020). However, Lira was able to restore the OPCs re-myelination ability via Olig2 transcription activation. On the contrary, administration of Dorso failed to significantly block the triggering effect of Lira on Olig2 protein expression. This reflects that Lira stimulates Olig2 via other multiple mechanisms. Results of brain weight, LFB-staining, MBP and Olig2 protein expression suggest that the mechanism by which Lira reverses Cup-induced neurotoxicity may be through its potential to induce re-myelination and prevent demyelination.

Upon activation of GLP-1R, it stimulates the phosphorylation of AMPK and activates the neuroprotective downstream signaling cascade (Carling et al. 2012). The current study clearly shows that Lira-treated group exhibited a significant increase in p-AMPK, which may explain the significant up-regulatory effect of Lira on SIRT1 suppression triggered by Cup intoxication (Peixoto et al. 2017). Previous research supports these findings (Elbaz et al. 2018; Houshmand et al. 2019), which revealed that Cup ingestion induced neurotoxicity via suppressing p-AMPK/SIRT1 markers. Moreover, Lira-modulatory effect is affirmed by Dorso co-administration as Lira + Dorso group showed significantly lower levels of p-AMPK and SIRT1 than Lira-treated group.

In line with its activation effect on AMPK/SIRT1 pathway, Lira markedly enhanced the autophagy machinery as verified by a significant increment in the autophagy biomarkers Beclin-1 and LC3B levels (Misrielal et al. 2020). In contrast, Cup was able to diminish the autophagic flux as shown in the reduced levels of Beclin-1 and LC3B, similar to what was formerly demonstrated (Dasgupta et al. 2013; Zhang et al. 2020). Based on the premise that autophagy activation participate in the regulation of mitochondrial homeostasis and neuroprotection (Kruppa et al. 2018; Misrielal et al. 2020), our findings depicted the suppressive effect of Lira on neuroinflammation through abolishing the Cup-induced mitochondrial damage and cellular debris via autophagy augmentation. This result is confirmed by the co-administration of Dorso as it lowered Beclin-1 and LC3B protein expression. Additionally, this may explain the significant decrement in HMGB1 level, as one of the most powerful damage-associated molecular patterns (DAMPs) (Shi et al. 2018), in Lira-treated group. In this context, we hypothesized that Lira may indirectly mitigate the activation of TLR-4 and subsequently NLRP3 inflammasomes via autophagy activation.

It is well established that Cup administration induces detrimental metabolic oxidative stress and primary OLGs apoptosis. A state that subsequently increases microglial activation and microgliosis significantly (Zatta et al. 2005; Blakemore and Franklin 2008; Hillis et al. 2016). It goes along with our results, as the Cup-treated group showed highly expressed Iba-1 in immunohistochemical pictures. However, Lira was able to mitigate the pathologic microgliosis as it showed a lower Iba-1 level.

The canonical NLRP3 activation is primed by the recognition of several DAMPs by the aid of pattern recognition receptors such as Toll-like receptors (TLRs) (Yuan et al. 2018). High mobility group box protein-1, a non-histone DNA-chaperone protein, is a key transcriptional modulator. It is released to the extracellular environment from the activated macrophages, monocytes, dendritic cells as well as apoptotic OLGs (Malhotra et al. 2015). Being a powerful DAMP, HMGB1 is detected by the surface TLR-4 and upregulates the transcription and activation of the inflammatory cytokine NF-κB. Thereafter, triggering NF-κB leads to the overexpression of inflammasome-related components such as NLRP3, pro-caspase-1, pro-IL-1β and pro-IL-18 as well (Agostini et al. 2004; Harris et al. 2011). Following the priming step, NLRP3 protein forms a heterogeneous oligomer with apoptosis-associated spec-like protein. Such oligomer, in turn, catalyzes the proteolysis of pro-caspase-1 into active caspase-1. This further gives rise to the formation and release of mature IL-1β and IL-18 (Ito et al. 2015; Birnbaum et al. 2018).

Cuprizone intoxication was able to provoke a heightened state of inflammation and apoptosis through elevating caspase-1 and IL-1β in brain tissue that is reflected in the histopathological pictures herein (Jha et al. 2010). The observed significant increase in HMGB1 in the Cup-treated group is attributed to the activation of immune cells and apoptotic OLGs, which is consistent with previous findings (Malhotra et al. 2015). In parallel, this was associated with upregulation of TLR-4 expression (Shi et al. 2018). The activation of GLP-1 resulted in inhibition of TLR-4 expression by the tested macrophages (Dai et al. 2014). More importantly, the current study shows the anti-inflammatory action of Lira via preventing Cup-induced overexpression of HMGB1 and TLR-4, indirectly and directly, respectively. Hence, it promisingly ameliorates the concomitant increases in NF-κB expression. These findings are in accordance with the literature data, which clearly showed that the activation of upregulated TLR-4 by overly expressed HMGB1 is assumed to trigger several cell responses. It markedly increased NF-κB level (Shi et al. 2018), as demonstrated in the Cup-treated group in our study. Accordingly, it may elucidate the overexpression of NLRP3, activated caspase-1 and activated IL-1β, as a downstream cascade, following Cup intoxication.

According to our findings, Lira treatment markedly attenuated the activation and release of the inflammatory cytokines such as caspase-1 and IL-1β via mitigating the Cup-induced NLRP3 overexpression. Indeed, IL-1β has been recognized as a significant contributor to the cellular apoptosis cascade (Cai et al. 2004; Prins et al. 2013). Therefore, the observed suppressive effect of Lira on IL-1β may contribute to its anti-apoptotic effect and consequently its anti-inflammatory effect.

Accumulating body of evidence presumed that both HMGB1/TLR-4 and AMPK/SIRT1 cascades tightly control NF-κB, which is a crucial transcriptional regulator of neuroinflammation (Liu et al. 2017). Moreover, previous studies reported the suppressive effect of both SIRT1 on NF-κB (Kuhlmann et al. 2008) and AMPK on TLR-4 expression (Soraya et al. 2012, 2014). In line with this, the current study depicted that Lira can mitigate neuroinflammation in Cup-treated mice through NLRP3 inflammasomes modulation both directly via GLP-1R and indirectly via AMPK/SIRT1 and autophagy, a finding that is verified by Dorso administration. Herein, Lira neuroprotective effect is partially abolished by pre-treatment with Dorso as it presented a significant decrement in HMGB1, TLR-4, and NF-κB levels in addition to NLRP3 expression and its downstream cascade, caspase-1, and IL-1β.

## Conclusion

In the current study, Lira exhibited neuroprotective effects against Cup-induced MS. The findings showed that Lira was able to restore the impaired behavioral and motor functions in Cup-treated mice. In addition, it revealed that Lira could activate autophagy, curtain inflammation, and suppress apoptosis. The current study also substantiates the possible interplay between AMPK/SIRT1, autophagy, and NLRP3 inflammasomes pathways and their role in the potential anti-inflammatory neuroprotective effect of Lira. At last, this study offers a new perspective for the use of Lira as a promising candidate in the management of MS and ameliorating its pathological consequences, especially for diabetic patients who suffer from multiple sclerosis co-morbidity. However, further investigations for its beneficial effects on humans through clinical trials are still needed.

## Data Availability

The datasets generated during and/or analysed during the current study are available from the corresponding author on reasonable request.
